# Consumption of Oleic Acid on the Preservation of Cognitive Functions in Japanese Elderly Individuals

**DOI:** 10.3390/nu13020284

**Published:** 2021-01-20

**Authors:** Keisuke Sakurai, Chutong Shen, Izumi Shiraishi, Noriko Inamura, Tatsuhiro Hisatsune

**Affiliations:** 1 Department of Integrated Biosciences, Graduate School of Frontier Sciences, The University of Tokyo, Kashiwa 277-8562, Japan; 3647306978@edu.k.u-tokyo.ac.jp (K.S.); 3032531168@edu.k.u-tokyo.ac.jp (C.S.); shiraishi@edu.k.u-tokyo.ac.jp (I.S.); 2 Community Health Promotion Laboratory, Mitsui Fudosan, Co., Ltd., Kashiwa 277-8519, Japan; inamura@udck.jp; 3 Urban Design Center Kashiwanoha (UDCK), Kashiwa 277-0871, Japan

**Keywords:** cognitive function, memory function, aging, fat, MUFA, oleic acid

## Abstract

We recruited 154 community-dwelling elderly individuals and conducted a cohort study to find out the nutrient intake that is suitable for maintaining cognitive function in Japanese elders. Cognitive function was evaluated by the two functional tests, the Montreal Cognitive Assessment (MoCA) and Wechsler Memory Scale-Delayed Recall (WMS-DR), and daily nutrient intake was estimated from a Brief-type Self-administered Diet History Questionnaire (BDHQ). By a multiple regression analysis, among the four major nutrients (protein, fat, carbohydrate and ash), we detected a significant correlation between the score of cognitive functions assessed by both MoCA and WMS-DR and daily consumption of fat (*p* = 0.0317 and *p* = 0.0111, respectively). Among categories of fatty acid, we found a significant correlation between the score of both MoCA and WMS-DR and consumption of monounsaturated fatty acid (MUFA) (*p* = 0.0157 and *p* = 0.0136, respectively). Finally, among MUFAs, we observed a significant correlation between the score of both MoCA and WMS-DR and consumption of oleic acid (*p* = 0.0405 and *p* = 0.0165, respectively). From these observations, we can propose that daily consumption of fat, especially in oleic acid, has a beneficial effect against cognitive decline in community-dwelling Japanese elderly individuals.

## 1. Introduction

The decline in cognitive function increases with aging, and age-related cognitive decline is one of major risk factors for the onset of dementia, such as Alzheimer’s disease (AD) [1,2,3,4,5]. The number of dementia patients is increasing not only in developed countries like European countries and the United States, but also in developing countries. In Asian countries including Japan, if the disease continues at the current rate, it is estimated that 67 million will have dementia by 2050, which is more than half of worldwide estimation of 130 million people [6]. In Western countries, research on prevention of dementia using diet patterns and specific nutrients has been widely performed. By large-scale epidemiological studies, nowadays, three dietary habituations have been proposed for the prevention of dementia. The Mediterranean diet pattern, such as the intake of olive oil and seafood, has been confirmed to be effective in preventing dementia [7,8,9,10]. Dietary Approaches to Stop Hypertension (DASH) is also effective in preventing dementia [7,8,9,10,11,12]. Furthermore, a research group at Rush University in Chicago, USA, led a proposal for their combination as Mediterranean-DASH Intervention for Neurodegenerative Delay (MIND) diet, which is a combination of these two diets, and confirmed its effectiveness in preventing dementia [13,14]. These dietary habits are mainly due to the fact that obesity is a problem even in old age in Europe and the United States. Therefore, it is recommended to avoid excessive calorie intake and to decrease the intake of red meat, dairy products, and fats and oils, mainly from the viewpoint of obesity control [7]. On the other hand, it is noteworthy that olive oil is listed as a food that should be consumed [10,15].

In contrast to European countries and the United States, in Asian countries, and especially in Japan, in old age, thinness rather than obesity is considered more of a problem [16,17]. The consumption of meat and dairy products is recommended to maintain health; however, there is still a great lack of systematic cohort data to direct this recommendation. Research on the causal interaction between preservation of cognitive function and daily nutrient intake pattern has not been systematically conducted for elderly people in East Asian countries such as in Japan, whose eating habits are different from those of Westerners. In recent years, it has been reported in Japanese elderly cohort studies, such as those of Hisayama and Osaki [18,19,20,21], that elderly people who consume a large amount of milk [18,19], meat [20] and tea [21] tend to have higher cognitive function. In addition, the results of intervention studies conducted by us have suggested that intake of functional dipeptides in meat [22,23,24,25,26] from chicken and fish, and vitamin K contained in matcha-green tea [27], is involved in the maintenance of cognitive function. However, there are not many studies that have investigated the relationship between oil intake and cognitive function, and so far there are no reports that have clarified the relationship between specific fatty acids and cognitive function.

In this study, to find out the nutritional components that are suitable for maintaining cognitive function in elderly Japanese people, we examined cohort study data of community-dwelling elderly Japanese people who lived in Kashiwa-city, one of the typical suburbs in Tokyo metropolitan Area. We utilized the BDHQ (Brief Self-administered Diet History Questionnaire) test [28], which is a questionnaire to calculate the estimated daily intake of all nutrients, including each fatty acid, from a daily food intake frequency survey. A multiple regression analysis was performed using the daily estimated nutrients’ intake based on the BDHQ as explanatory variables, and using two types of cohort cognitive function indicators as independent objective functions.

## 2. Materials and Methods

### 2.1. Participants

**Figure 1:**
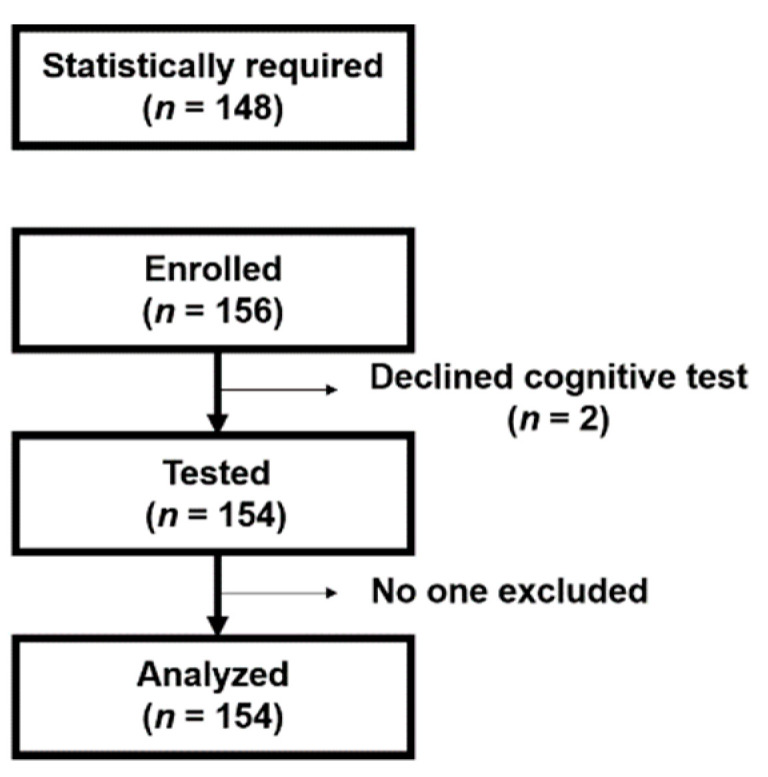
The flow chart of participants’ recruitment.

We designed this study based on the assumption by a Cohen’s *f^2^* [29], which consisted of an effect size of 0.15, a type 1 two-sided error protection of 0.01, and 80% of the power. The number of subject samples required was calculated to be 148. We recruited 154 elderly individuals living in Kashiwa-city, Japan (Figure 1). Based on the age cutoffs defined by the United Nations [30], we recruited individuals over the age of 60 as elderly people. Those previously diagnosed in clinic with mild cognitive impairment (MCI) or dementia were excluded from the study. All participants had submitted the written informed consent in advance for inclusion. Our research was conducted in accordance with the Declaration of Helsinki, and the protocol was approved by the Ethics Committee of the University of Tokyo.

A total of 154 community-dwelling elderly people underwent a nutritional consumption survey and cognitive function tests, and data from all subjects were used in the analysis.

### 2.2. Outcome Measures

Cognitive function in participants was evaluated by the two psychometric tests. The Montreal Cognitive Assessment (MoCA) test [31] is somewhat more difficult than the Mini-Mental State Examination (MMSE) test, which has been widely used for cognitive screening. Because of its high level of difficulty, the MoCA test has high sensitivity and specificity for detecting MCI compared to MMSE [31]. The Wechsler Memory Scale-Delayed Recall (WMS-DR) test [32] is a test of verbal memory using narrative sentences. We used the WMS-DR to evaluate logical memory function.

### 2.3. Assessment of Dietary Habits

To estimate the consumption of each nutrient for all subjects, we assessed their dietary habits. Dietary habits vary by culture; therefore, it is necessary to select a survey of eating habits suitable for each country. In this study, we used the BDHQ developed by Kobayashi et al. [28], which is suitable for Japanese dietary habits, and widely applied in evaluating dietary habits in Japan. In this questionnaire, the calculation of the intake amount of typical nutrients takes, as a reference, the Standard Tables of Food Composition in Japan [33] by filling in the average frequency and intake amount of each food.

### 2.4. Statistical Analysis

To evaluate the association between the dietary intake profile and the cognitive/memory function, we conducted a multiple linear regression analysis with MoCA and WMS-DR scores as objective variables, and the estimated daily consumption of each nutrient based on BDHQ as explanatory variables. We included the explanatory variables in the step-wise multiple linear regression analysis if the significance was *p* ≤ 0.20 for the primary outcome. If explanatory variables remained statistically significant at a level of *p* ≤ 0.20, these were incorporated into the final multiple regression model. We defined a *p*-value of less than 0.05 as statistically significant. In the primary analysis of multiple regression, we selected the consumption of four major nutrients (protein, fat, carbohydrate and ash) as exploratory variables. In the secondary analysis of multiple regression, we chose the consumption of the categories of fatty acid (Saturated fatty acid (SFA) and Mono-unsaturated fatty acid (MUFA), n-3 fatty acid (N3), and n-6 fatty acid (N6)) as exploratory variables. In the tertiary analysis of multiple regression, we classified the consumption of the categories of MUFA (palmitoleic acid (16:1) and oleic acid (18:1)) as exploratory variables.

The variables used as candidates for covariates incorporated into the final multiple regression model were age, sex, body mass index (BMI), and years of education. Finally, the combination of covariates with the lowest Akaike’s information criterion was adopted as the final model [34]. Even those who do not have dementia or MCI may experience subtle cognitive decline with aging [35], and it has been reported that there are gender differences in cognitive changes associated with aging in clinically normal elderly people [36]. Sex is coded as 1 = Male, 2 = Female. Besides, quality of education impacts late-life cognition [37]. Although findings from previous studies on the relationship between cognitive function and BMI in the elderly are inconsistent, we adopted BMI as a candidate for covariates because some studies conclude that BMI in the elderly is associated with cognitive changes [38,39].

According to Hair et al., the Variance Inflation Factor (VIF) is considered problematic for multicollinearity when the value is over 10 [40]. Since there was no factor with a VIF over 10 in our present study, no multicollinearities between the explanatory variables were taken into consideration.

## 3. Results

### 3.1. Participants

A total of 154 Japanese community-dwelling individuals were recruited (50 men, 104 women). The inclusion criteria for age ranged from 60 to 84 years (average, 73.1 years). Among all participants, BMI ranged from 15.6 to 31.8 kg × m^−2^ (average, 22.1 kg × m^−2^), and years of education ranged from 9 to 21 years (average, 13.7 years) (Table 1).

### 3.2. Multiple Linear Regression Analyses with the Consumption of Nutrients

We conducted multiple regression analyses to detect the association between cognitive function and the consumption of nutrients (Table 2, Table 3, Table 4, Table 5, Table 6 and Table 7). Multiple linear regression was performed with the cognitive functions assessed by either the MoCA score or the WMS-DR score as the objective variable, and with the consumption of nutrients as explanatory variables. Age, sex, years of education, and BMI were incorporated into the multiple regression model as candidates for covariates. Table 2, Table 3 and Table 4 describe the results of a multiple linear regression that represents the association between MoCA score and the consumption of nutrients. Table 5, Table 6 and Table 7 shows the results of a multiple linear regression that examines the association between WMS-DR score and the consumption of nutrients.

As a result of multiple regression analysis with the MoCA score as the objective variable and the consumption of protein, fat, carbohydrate and ash as the explanatory variables, a significant regression equation was found (*R*^2^ = 0.13, *p* = 0.0004). Age and the consumption of fat are significant predictors of the MoCA score (*p* = 0.0141 and *p* = 0.0317, respectively).

As a result of multiple regression analysis with the MoCA score as the objective variable and the consumption of SFA, MUFA, N3 and N6, a significant regression equation was found (*R*^2^ = 0.13, *p* = 0.0003). Age and the consumption of MUFA are significant predictors of the MoCA score (*p* = 0.0055 and *p* = 0.0157, respectively).

As a result of multiple regression analysis with the MoCA score as the objective variable and the consumption of palmitoleic acid and oleic acid, a significant regression equation was found (*R*^2^ = 0.13, *p* = 0.0005). Age, years of education and the consumption of oleic acid are significant predictors of the MoCA score (*p* = 0.0189, *p* = 0.0456 and *p* = 0.0405, respectively).

As a result of multiple regression analysis with the WMS-DR score as the objective variable and the consumption of protein, fat, carbohydrate and ash, a significant regression equation was found (*R*^2^ = 0.20, *p* < 0.0001). Age, years of education and the consumption of fat are significant predictors of the WMS-DR score (*p* = 0.0018, *p* = 0.0004 and *p* = 0.0111, respectively).

As a result of multiple regression analysis with the WMS-DR score as the objective variable and the consumption of MUFA, N3, and N6, a significant regression equation was found (*R*^2^ = 0.20, *p* < 0.0001). Age, years of education and the consumption of MUFA are significant predictors of the WMS-DR score (*p* = 0.0024, *p* = 0.0004 and *p* = 0.0136, respectively).

As a result of multiple regression analysis with the WMS-DR score as the objective variable and the consumption of palmitoleic acid and oleic acid, a significant regression equation was found (*R*^2^ = 0.19, *p* < 0.0001). Age, years of education and the consumption of oleic acid are significant predictors of the WMS-DR score (*p* = 0.0026, *p* = 0.0004 and *p* = 0.0165, respectively).

## 4. Discussion

This cohort study is the first to clearly show the positive effects of oleic acid on cognitive function in community-dwelling elderly individuals. The reasons we can insist on this observation are because (1) multiple regression analysis showed statistically significant effects on two independent measures of psychological functioning (MoCA and WMS-DR); (2) stepwise multiple regression analysis showed that MUFA among the saturated fatty acid types and fat among the four nutrients had statistically significant effects on two independent measures of psychological functioning—MoCA and WMS-DR; and (3) the fact that these results were obtained in elderly people in Japan, where the intake of fat is relatively low compared to the Western countries. If a similar study were conducted in Europe or the United States, it would be difficult to imagine that the effects of obesity would lead to positive effects of fat intake on cognitive function, and indeed some negative effects have been found. Therefore, if the first step of the multiple regression analysis did not show that fat intake was good, the subsequent stepwise analysis of oleic acid could not have been conducted.

In this study, we utilized two psychological tests. MoCA has been suggested to be a more valuable tool for screening mild levels of cognitive dysfunction in AD and MCI [31,41], and was used to evaluate general cognitive function of elderly individuals. The WMS-DR is a subordinate category of WMS, which evaluates episodic memory function by testing the immediate and delayed recall on a meaningful story presented verbally [42]; therefore, we evaluated the episodic memory function of our participants by WMS-DR score. The two tests are mutually independent, and while delayed replay is highly sensitive to memory impairment in healthy elderly people, the MoCA test is more sensitive than delayed replay in discriminating MCI, which are the prodromal stages of dementia. Judging from this fact, we can conclude that oleic acid has an effect on both of the brain functions that these tests are assessing or reflecting.

As for the results on the relationship between diet pattern and the cognitive function, a positive correlation between the daily intake amount of fat and the score of cognitive function was shown, which may suggest that the fat profile in our daily life may have a potential effect on the cognitive function. However, it is known that different categories of fat may have totally different effects on health. Thus, in order to properly evaluate the actual effective categories of fat, we also conducted the multiple regression analysis separately on the effect of different categories of fatty acid and the score of cognitive function tests. Consequently, we found that analysis of MUFA showed a significantly positive effect toward the score of cognitive tests, and especially in the group of oleic acid, a significantly positive effect was also found. Thus, we assumed that the MUFA, especially the sources from oleic acid, might be one of essential nutrients to the maintenance of cognitive function.

Fatty acids (FAs) are an important component of the diet and play a major role in the lipid composition of cell membranes [43]. Some fatty acid metabolites, such as prostaglandins, thromboxanes and leukotrienes, also play a crucial role as cell signaling molecules [44]. Besides, among various types of fatty acids, omega-3, omega-6, and omega-9 fatty acids have been recognized to act as neuroactive molecules [45]. For instance, administration of omega-3 PUFAs has been assessed for the treatment of neurodegenerative disease and Alzheimer’s disease [46,47]. As for the omega-6 fatty acid, it is known to be beneficial in the prevention of inflammation, free radical production, or even the neurological deterioration [48]. With regard to omega-9 fatty acids, oleic acid is one of the most representative monounsaturated fatty acids, which is usually considered to be beneficial in anti-inflammatory and vascular activities [49]. Some dietary patterns are well-known for containing high amounts of this kind of monounsaturated fatty acid and have been highly been proposed to link with the anti-aging effect, including the improvement in cognitive function. For instance, the Mediterranean diet, one of the most representative dietary patterns often used in the Southern Europe, is highly recognized as being linked with the protection against age-related cognitive decline, and the notable effects may be related to the presence of high amounts of oleic acid [50,51,52]. In addition, though the Mediterranean diet is not as popular as it is in Europe, there is a similar diet pattern in Asia, the “Okinawa diet”, which also features high contents of monosaturated fatty acid and has been claimed to have extensive anti-aging effects [53]. Thus, this evidence has indicated the importance of monounsaturated fatty acid, especially oleic acid, for its effect on anti-aging or on brain health.

On the other hand, these similar effective benefits were also shown in animal studies, in which administration of oleic acid in a rodent model shown its neuroprotective effect in transient and permanent focal cerebral ischaemia, as well as against global cerebral ischaemia-induced delayed neuronal death [54]. Although the underlying mechanisms between oleic acid and cognitive function have not been well elucidated, it is believed that the anti-inflammatory and vascular activities that oleic acid is associated with are related to the onset of neurodegeneration disease [49]. Besides, the dysregulation of the enzyme stearyl coenzyme A desaturase-I (SCD-1), which is responsible for oleic synthesis within the central nervous system, has been shown to act as a possible driving force to the Alzheimer’s disease pathology [55]. Moreover, in the oleic acid supplementation in transgenic mice featured in Alzheimer’s disease pathology, it leads to the reduction in the secreting Aβ levels in amyloid precursor protein (APP) 695 transfected Cos-7 cells, but also the reduction in BACE-1 levels and presenilin levels, and has caused the amyloid plaques in the brain to reduce [56]. Therefore, it is highly likely that oleic acid may be a beneficial nutrient that is closely related to neurological diseases. Likewise, in our present study, we also revealed, as a consistent result, that either oleic acid or the group of the MUFAs showed a significantly positive effect on the scores of cognitive functions. Thus, it may strengthen the beneficial role of oleic acid in cognitive function, which may also potentially contribute to a recommend dietary pattern toward the prevention of cognitive dysfunction. Still, a further elucidation of the mechanisms underlying the oleic acid and the cognitive function is needed in future research.

Several limitations of our study need to be considered. First, the population size is a limitation in our present study, which may not provide adequate sensitivity in the detection of cognitive function in tests, including the score of the MoCA test. Thus, future study with a larger population size is needed to amplify the insights in our present findings. Second, since BDHQ evaluates nutritional consumption based on the intake of 50 types of foods, which is a typical Japanese diet, it may not be possible to properly evaluate it in subjects with special eating habits. Finally, since BDHQ is a self-reported evaluation method, the estimated nutritional consumption may differ from the actual intake. Therefore, quantification of food intake by an observer is needed when conducting future studies investigating the relationship between more accurate nutritional consumption and cognitive functions.

## 5. Conclusions

In the present study, we conducted a multiple regression analysis of cohort study data to find out the food nutrients suitable for maintaining cognitive functions among Japanese community-dwelling elderly. It was suggested that fat intake, especially oleic acid intake, may be effective for both cognitive and memory functions. It is notable that this is the first study to point out the importance of fatty acid intake in maintaining brain function in the elderly in East Asian areas such as Japan.

## Figures and Tables

**Table 1:** Participant characteristics.

Item	Value
Age *	73.1 ± 5.0
BMI *	22.1 ± 2.7
Years of Education *	13.7 ± 2.2
Sex (Male/Female) ^#^	50/104

* Data are mean ± standard deviation. ^#^ Data are described as the number of individuals. BMI: body mass index.

**Table 2:** Association between the Montreal Cognitive Assessment (MoCA) score and consumption of fat.

Variable	MoCA				
	B	SE B	β	f	*p*
Age	−0.12	0.05	−0.20	6.17	0.0141
Sex	0.79	0.55	0.13	2.09	0.1505
Years of Education	0.23	0.11	0.17	3.91	0.0500
Fat	53.6	24.7	0.17	4.70	0.0317
*R* ^2^	0.13				
F	5.48				
*P*	0.0004				

Β: Partial regression coefficient. SE B: Standard error of the partial regression coefficient. β: Standardized partial regression coefficient. f: F value of the statistical significance in the partial regression coefficient. *p: p* value of the statistical significance in the partial regression coefficient. *R*^2^: Coefficient of determination of multiple linear regression equation. F: F value of the statistical significance in multiple linear regression equation. *P*: *p* value of the statistical significance in multiple linear regression equation.

**Table 3:** Association between MoCA score and consumption of monounsaturated fatty acid (MUFA).

Variable	MoCA				
	B	SE B	β	f	*p*
Age	−0.13	0.05	−0.22	7.94	0.0055
Years of Education	0.18	0.10	0.14	3.05	0.0827
MUFA	400	163	0.46	5.97	0.0157
N6	−499	309	−0.31	2.62	0.1079
*R* ^2^	0.13				
F	5.66				
*P*	0.0003				

MUFA: monounsaturated fatty acid. N6: n-6 fatty acid.

**Table 4:** Association between MoCA score and consumption of oleic acid.

Variable	MoCA				
	B	SE B	β	f	*p*
Age	−0.11	0.05	−0.19	5.63	0.0189
Years of Education	0.23	0.11	0.17	4.06	0.0456
Sex	0.84	0.55	0.14	2.36	0.1266
Oleic Acid	0.16	0.08	0.16	4.27	0.0405
*R* ^2^	0.13				
F	5.36				
*P*	0.0005				

**Table 5:** Association between Wechsler Memory Scale-Delayed Recall (WMS-DR) score and consumption of fat.

Variable	WMS-DR				
	B	SE B	β	f	*p*
Age	−0.20	0.06	−0.24	10.1	0.0018
Years of Education	0.51	0.14	0.27	12.9	0.0004
BMI	0.19	0.11	0.12	2.75	0.0995
Fat	86.1	33.5	0.19	6.61	0.0111
*R* ^2^	0.20				
F	9.29				
*P*	<0.0001				

**Table 6:** Association between WMS-DR score and consumption of MUFA.

Variable	WMS-DR				
	B	SE B	β	f	*p*
Age	−0.20	0.06	−0.23	9.56	0.0024
Years of Education	0.51	0.14	0.27	13.33	0.0004
BMI	0.19	0.12	0.12	2.56	0.1120
MUFA	228.7	91.6	0.19	6.23	0.0136
*R* ^2^	0.20				
F	9.18				
*P*	<0.0001				

**Table 7:** Association between WMS-DR score and consumption of oleic acid.

Variable	WMS-DR					
	B	SE B	β	f	*p*
Age	−0.19	0.06	−0.32	9.37	0.0026
Years of Education	0.51	0.14	0.27	12.93	0.0004
BMI	0.18	0.12	0.12	2.45	0.1196
Oleic Acid	0.25	0.10	0.18	5.88	0.0165
*R* ^2^	0.19				
F	9.07				
*P*	<0.0001				

## Data Availability

Data can be obtained by contacting the corresponding author.
